# An 11-year-old boy with immediate allergic reaction to articaine but not to lidocaine

**DOI:** 10.1186/2045-7022-4-S3-P71

**Published:** 2014-07-18

**Authors:** Wasu Kamchaisatian, Nopalit Insorn, Nattipat Juthacharoenwong, Surangkana Techapaitoon

**Affiliations:** 1 Samitivej International Children's Hospital, Bangkok Hospital Group, Allergy and Immunology Center, Thailand; 2 Samitivej International Children's Hospital, Bangkok Hospital Group, Pediatric Center, Thailand

## Background

Adverse reaction to local anesthetic agents is relatively common, but true IgE-mediated hypersensitivity is rare. Allergic reaction to these drugs can be harmful especially in the circumstance with non-allergy physician i.e. dentist performing dental procedure which may cause severe systemic reaction.

## Objective

To report a boy with immediate local reaction to articaine, a local anesthetics widely used in dental procedure, with demonstrable IgE antibody to articaine, but not to lidocaine.

## Case report

An 11 years old boy had history of lip swelling and itching after dental procedure with injection of local anesthetic drug, Articaine (Ubistesin®). There was no any skin rash or hives on any parts of the body and no other systemic symptoms. Lip swelling lasted for 2 hours and then spontaneously resolved. The patient was consulted for suspicious allergy to articaine. After thoroughly history taking, he also has allergic rhinitis and previous history of asthma, but no any allergy to latex or drugs including sulfonamide. Skin prick test and intradermal test were done with undiluted and 1/10 dilution of the following amide local anesthetics in a single-use dental cartridge of: articaine, lidocaine, mepivacaine and levobupivacaine; but not to procaine, the ester local anesthetics, since it was unavailable. The results revealed markedly positive to intradermally testing of undiluted and 1/10 dilution of articaine and mepivacaine, but negative to lidocaine and levobupivacaine. We did not perform provocative dose challenge to lidocaine or levobupivacaine, however, subsequently; the patient was undergone dental procedure using lidocaine as the local anesthetics with no any local or systemic reaction.

## Conclusion

When allergic reaction to local anesthetics is suspected, an allergy consultation for skin testing and provocative dose challenge should be appreciated. This may help confirming the suspected culprit agent that may be safely used, or moreover, to identify a suitable alternative.

